# Healthy Eating Determinants: A Study among Malaysian Young Adults

**DOI:** 10.3390/foods9080974

**Published:** 2020-07-23

**Authors:** Abdullah Al Mamun, Naeem Hayat, Noor Raihani Binti Zainol

**Affiliations:** 1 UCSI Graduate Business School, Faculty of Business and Management, UCSI University, Cheras, Kuala Lumpur 56000, Malaysia; 2 Faculty of Entrepreneurship and Business, Universiti Malaysia Kelantan, Kota Bharu 16100, Malaysia; naeem.a18e013f@siswa.umk.edu.my (N.H.); raihani@umk.edu.my (N.R.B.Z.)

**Keywords:** health consciousness, knowledge about healthy food, theory of planned behaviour, intention and behaviour, healthy food, Malaysian young adults

## Abstract

This study aimed to examine the effect of health consciousness, knowledge about healthy food, attitudes toward healthy food, subjective norms, and perceived behavioural control on the intention to consume healthy food, which subsequently affects the consumption of healthy food among Malaysian young adults. The current study also examined the moderating effect of perceived barriers on the association between intention to consume healthy food and the consumption of healthy food. This study adopted a cross-sectional design and collected quantitative data from 1651 Malaysian young adults (between the age of 18 and 40 years) by sharing a Google form link through social media. The findings reveal that health consciousness, knowledge about healthy food, attitude toward healthy food, subjective norms, and perceived behavioural control had a significant positive effect on the intention to consume healthy food. Findings also show that the intention to consume healthy food has a significant positive effect on the consumption of healthy food among Malaysian young adults. Furthermore, the findings reveal the positive and significant mediating effect of the intention to consume healthy food and the significant moderating effect of perceived barriers on the association between the intention to consume healthy food and the consumption of healthy food. The multi-group analysis revealed that the effect of perceived barriers on the consumption of healthy food and the moderating effect of perceived barriers were significantly higher among urban respondents. Health and agriculture policymakers should focus on the attributes of healthy eating practices and their health benefits to promote the mass adoption of healthy food among Malaysian young adults.

## 1. Introduction

Modern society is confronting increased health issues as the population’s eating habits and the lack of healthy food consciousness had caused obesity and poor nutrition and eating conditions among young adults [1]. About 2.8 million people die worldwide because due to being overweight or having melancholic obesity each year [2]. Since 2000, Malaysians are facing issues of obesity and eating disorders [3]. Diet-related diseases are on the rise in Malaysia, and this is increasing the socioeconomic burdens on middle-income households [1]. Scientific evidence shows that unhealthy and unbalanced food increases the risk of hypertension, cardiovascular diseases, and diabetes [4]. Whole grains, fruits, vegetables, and legumes are essential for a healthy life besides reducing certain medical conditions [5].

Thirty-nine percent of the world population is overweight, and about 13% of the population is obese [3]. Malaysians are the most obese citizens in Southeast Asia, in which 48% of the population is experiencing obesity [1]. Lifestyle changes and modern lifestyles make life more comfortable, and food security improves the dietary intake among the middle class and upper class of the developed and developing nations [3,6]. Poor eating habits and insufficient physical activities are causing obesity and non-communicable diseases [7]. Healthy food consciousness is on the rise among young adults at the global level [8]. The improved awareness of healthy food promotes the addition of nutritional labelling on food and food menus by food sellers [9]. Restaurants provide information on food calories and serve food-conscious customers by charging premium prices.

The concern for healthy food has increased from the year 2000, and the health problems among global youth have increased in recent time [10]. Healthy food is gaining attention and interest from the food industry and policymakers. Food industry players improve the food, and policymaker drafted specific guidelines to provide relevant food-related information [7]. Customers who have a more significant concern for health are more inclined to consume healthy food even at premium prices [5]. The provision of food-related information from the foodservice providers can improve customer satisfaction and food business [11].

Having a healthy eating lifestyle is on the rise, and it reduces health risks while improving the lives of the population [3]. Southeast Asians are known for having a higher number of obese people in the world as they have unhealthy eating habits and lifestyle [3]. A further reason for the low adoption of healthy food is the price [12]. For instance, low-energy food is more affordable compared to food with high energy content and is a determining factor, similar to price, toward the adoption of healthy food. While the Malaysian government supports and promotes a healthy lifestyle [1], the adoption of healthy eating habits remains at an initial stage of adoption amongst Malaysians. In contrast, consumer awareness and government support regarding food prices can help to improve the acceptance of healthy food consumption amongst Malaysian young adults.

Similarly, unhealthy eating habits are influenced by psychological factors like attitude [4]; the perception of barriers or benefits [3,6]; social factors like perceived support, behaviour, social influence [3,13]; and environmental factors like accessibility to healthy food and price [5,12].

Poor eating habits and lack of physical activities among Malaysians can enhance an unhealthy lifestyle, and the Malaysian national food policies are inadequate [3]. Unbalanced energy intake is high among Malaysians, and causes inadequate dietary quality that can increase the risk of medical conditions [1]. The remedy is using healthy and balanced dietary practices [3]. This study aims to explore the intention to consume healthy food and the consumption of healthy food among Malaysian young adults by the theory of planned behaviours (TPB). It also extended the TPB by health consciousness and knowledge of healthy food, and consumption behaviour is affected by perceived barriers.

The subsequent section of the paper deliberates on pertinent works and the development of the hypotheses. The next section presents the summaries of the method, followed by the analysis and results. The last section provides a discussion and conclusion.

## 2. Literature Review

### 2.1. Theoretical Foundation

One of the prime pertinent theories that explore human behaviour is TPB. TPB considers the attitudes toward specific behaviour, which is associated by the prevailing subjective norm about that behaviour, and the perceived behavioural control formulates the intention to behave in a particular manner and intention that leads to the specific behaviour [14]. The rule of thumb is that the favourable attitude, subjective norms, and higher perceived control can develop a firm intention to behave in a specific manner [15]. Furthermore, the behaviour can strongly be influenced by the intention toward the behaviour [14]. TPB is extensively utilised for predicting the intention and consumption behaviour for the environmental product and health-related behaviours [5,15]. Several studies explored the intention and consumption of healthy foods. Individual attitude, subjective norm, and perceived behavioural control can significantly influence the intention to consume healthy foods [5].

### 2.2. Consumption of Healthy Food (CHF)

Healthy foods are gaining higher acceptability among the general population in recent times [8]. Factors that can lead to higher acceptability are the more significant concern for personal health, a higher rate of obesity among young adults, and increasing health disorders [1,3]. Individuals that have the understanding and knowledge of healthy food can improve the intention to use healthy foods [4]. Moreover, the concern of personal health can help to formulate healthy eating habits. Governmental agencies are increasingly reporting upsurges in health ailments among young adults [3]. Moreover, advancements in nutrition research enable a better understanding of the human body’s nutritional requirements [4]. The healthy balance lifestyle relies on sensible daily food based on dietary recommendations and guidelines. Young consumers are willing to use conventional and newly developed food supplements to achieve the benefits of food, and they are willing to pay premium prices for nourishment food products [3,7].

Healthy foods improve individual health. Healthy foods provide adequate nutritional ingredients that reduce disease risks and improve health issues among individuals [1]. Healthy foods are a mix of commonly available food that have health-related beneficial effects on human health [11]. Healthy food has nutritional and physiological effects on the human body [3]. Health-related food offerings are increased in Malaysia, and the percentage of healthy food accounts for about 40% of total food offerings [10].

### 2.3. Factors Affecting the Intention to Consume Healthy Food

#### 2.3.1. Health Consciousness (HTC)

Health consciousness (HTC) is the perceived importance of health in an individual’s daily life routines. It reflects the individual’s willingness to adopt a healthy routine, food, and lifestyle [13]. Healthy food is indispensable for a healthy lifestyle and provides the necessary minerals and proteins to boost health and reduce the risk of diseases (6]. HTC is vital for a healthy life. An individual’s health consciousness can significantly affect the intention to use healthy foods [4]. Singh and Verma [6] postulated that HTC positively and significantly (β = 0.18, *p* = 0.01) influences the intention to consume healthy food (IHF) among Indian consumers.

**Hypothesis 1** **(H1).** *HTC has a significant positive effect on IHF among Malaysian young adults*.

#### 2.3.2. Knowledge about Healthy Food (KHF)

Knowledge is recognised as a critical factor for human behaviours. Food knowledge influences an individual’s eating behaviours [6]. Food-selecting behaviour is influenced by product knowledge, and knowledge enhances product understanding and healthy food behaviour [11]. Low levels of healthy food knowledge demonstrate poor eating behaviours among young adults [5]. Consumer awareness and knowledge can develop the intention to use environmentally friendly products and innovations [6]. Lee et al. [11] postulated that KHF significantly (β = 0.297, *p* = 0.000) influence the intention to use healthy food among Korean adults.

**Hypothesis 2** **(H2).** *KHF has a significant positive effect on the IHF among Malaysian young adults*.

#### 2.3.3. Attitude toward Healthy Food (AHF)

Attitude represents the overall evaluation of the perceived consequences of particular behaviour under the consideration of an individual [14]. A positive attitude toward behaviour can guide the intention to perform that behaviour [15]. Rezai et al. [10] postulated that ATFs positively and significantly (β = 0.116, *p* = 0.000) influences IHF among the Malaysian sample. Moreover, Nguyen et al. [8] reported that attitude toward functional food (β = 0.353, *p* = 0.000) positively affects the intention to purchase function food among Vietnamese youth. Hence, this study proposed the following hypothesis:

**Hypothesis 3** **(H3).** *AHF has a significant positive effect on IHF among Malaysian young adults*.

#### 2.3.4. Subjective Norms (SBNs)

Subjective norms (SBNs) are the perceived social pressure on an individual to perform or not to perform certain behaviours [14]. Perception of social pressure induces the social behaviours of individuals. Rezai et al. [10] postulated that subjective norm positively and significantly (β = 0.198, *p* = 0.000) influences the intention to purchase healthy food among Malaysian consumers. Furthermore, Menozzi et al. [5] postulated that SBN significantly (β = 0.56, *p* = 0.000) influences the intention to use green food among Italian students. Therefore, the following hypothesis is proposed:

**Hypothesis 4** **(H4).** *SBNs have a significant positive effect on IHF among Malaysian young adults*.

#### 2.3.5. Perceived Behavioural Control (PBC)

Individual perception of ability effects the performance of the behaviour [10]. Perceived behavioural control (PBC) is an individual’s understanding of the ease or difficulty associated with the performance of a behaviour. PBC affects the intention of green behaviours [15]. Menozzi et al. [5] postulated that PBC significantly (β = 0.69, *p* = 0.000) influences the intention to use green food among Italian students. Therefore, the following hypothesis is proposed:

**Hypothesis 5** **(H5).** *PBC has a significant positive effect on IHF among Malaysian young adults*.

#### 2.3.6. Intention to Consume Healthy Food (IHF)

Intention is the first outcome of the TPB based on attitude, SUN, and PBC [14]. There is empirical evidence that intention leads to the consumption behaviours [15]. Nguyen et al. [8] postulated that PBC significantly (β = 0.35, *p* = 0.000) influences CHF among young Vietnamese consumers. This study proposed the following hypothesis:

**Hypothesis 6** **(H6).** *IHF has a significant positive effect on CHF among Malaysian young adults*.

### 2.4. Mediating Effect of the Intention to Consume Healthy Food

Intention is the integral outcome of the TPB that leads to a particular behaviour. Singh and Verma [6] reported that intention mediates the three factors of TPB for the consumption behaviours for healthy organic food among Indian consumers. Moreover, the current work expanded the TPB with the factors of HTC and KHF. This study proposed the following hypothesis:

**Hypothesis 7** **(H7).** *IHF mediates the relationship between HTC, KHF, ATF, SUN, and PBC on CHF among Malaysian young adults*.

### 2.5. Moderating Effect of Perceived Barriers (PBS)

Healthy foods are perceived as beneficial and good for health. Instead of having HTC and KHF, the CHF is scant [3]. Perceived barriers (PBS) restrict CHFs [10]. Healthy foods are perceived as the difficulty to find, cook, and eat [6]. These PBS reveal the individuals’ belief that healthy food is costly, difficult to procure, and time-consuming to cook [7]. PBS have significant unfavourable effects on the intention to consume healthy foods [3]. Rezai et al. [10] postulated that PBS significantly (β = −0.083, *p* = 0.000) reduces the IHF among the Malaysian sample. Higher intention leads to higher consumption behaviour toward healthy food. PBS have adverse effects on the consumption behaviour of healthy food. This study, therefore, examined the moderating effect of PBS between the IHF and the CHF. Hence, the following hypothesis is proposed:

**Hypothesis 8** **(H8).** *PBS moderate the relationship between IHF and CHF among Malaysian young adults*.

**Figure 1:**
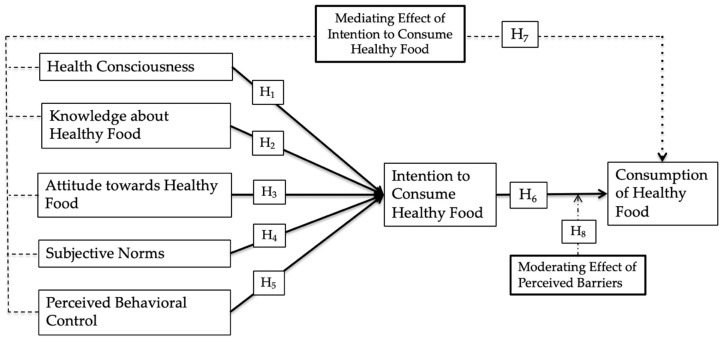
Research framework.

All association hypothesized and tested associations are presented in Figure 1.

## 3. Research Methodology

### 3.1. Data Collection and Study Sample Design

This study examined the effect of HTC, KHF, ATF, SUN, and PBC on IHF, which subsequently affects CHF among Malaysian young adults. In Malaysia, “youth” can be defined as those aged between 15 and 40 years old [16]. However, in order to avoid ethical issues and/or parental permission requirements for the collection of data, those aged below 18 years were excluded from this study, with those aged between 18 and 40 years meeting the criteria for Malaysian young adults. This study adopted the cross-sectional design and collected quantitative data from 1651 Malaysian young adults through an online survey for the first two weeks of April 2020. This study designed a Google form, highlighted the purpose, reported the procedure of the study, and collected informed consent from all respondents before they participated in the survey. The questionnaire was distributed by sharing the link of the questionnaire form using social media.

### 3.2. Survey Instrument

Explicit and straightforward statements were designed to gauge responses to the given constructs. This approach can obtain an appropriate and accessible understanding of the survey respondents. A total of five questions measuring HTC were adopted from several studies [7,8]. This study measured KHF using five questions adopted from several studies [13,17]. Five questions measured AHF adopted from several studies [5,10]. Five questions were adopted from several studies to measure SUN [7,10]. Five questions to measure PBC were adopted from several studies [5,15]. Five questions were adopted from several studies to measure PBS [10,13]. Four questions measuring IHF were adopted from several studies [5,10]. One question was adopted from a study by Menozzi et al. [5] to measure the CHF. All question items were assessed against a 7-point Likert scale, except for CHF, as this was measured as ‘yes’ or ‘no’. All questions are presented in Appendix A.

### 3.3. Assessment of Common Method Variance (CMV)

CMV issue is normal in social science research due to the data collection methods and techniques [18]. Harman’s [19] one-factor test was suggested to estimate the impact of CMV on study constructs [18]. One-factor Harman’s test revealed that CMV was not a critical matter for study, as the main factor accounted for 31.84% variance and less than the recommended limit of 50% [18].

### 3.4. Multivariate Normality

SEM-PLS is not associated with multivariate normality in the data, as it is a non-parametric analysis instrument [20]. Multivariate data normality was tested as suggested by Peng and Lai [21] using an online tool of web power (https://webpower.psychstat.org/wiki/tools/index) to confirm data normality. The test results confirm that the data set is not as normal as Mardia’s multivariate coefficient *p*-values that are less than 0.05 [22].

### 3.5. Data Analysis Method

Partial least squares structural equation modelling (PLS-SEM) was used with Smart-PLS software 3.1 for data analysis. PLS-SEM is a multivariate analysis instrument used to gauge the path models that have latent constructs with composites [20]. PLS-SEM empowers the researcher to tackle non-normal and small data sets. Furthermore, PLS-SEM has a casual-predictive nature with an undisturbed supposition of goodness-of-fit estimation compared to covariance-based SEM [23]. Two-step techniques analysed data with PLS-SEM, and the first measurement was performed to test the model’s reliability and validity at the constructs’ level [20]. The second stage was executed for the estimation of the structural model and the investigation of study hypotheses with significance levels [23]. Model estimation was performed with *r*^2^, Q^2^, and the effect size *f*^2^ that describe the path effect from the exogenous construct for the endogenous construct [20].

Multi-group analysis (MGA) in PLS-SEM permits the researchers to distinguish the differences between the pre-defined groups [24]. MGA is a convenient procedure to evaluate the differences between the groups inside the data set [20]. The MGA evaluates the distinctions among the structural paths of several groups in the data sets [24]. MGA was performed with the development of groups within data based on the categorical variables of interest like age, gender, or income. Then, the path coefficients for the groups were analysed whether two groups were significantly different from each other or not based on the procedures suggested by Henseler et al. [24]. The differences within the data set were based on the characteristics of samples that may not be noticeable in the collected data. Path coefficients of the group data can confirm the statistical variance using MGA to establish significant statistical differences among data based on categorical bases [24].

Importance-performance map analysis (IPMA) categorises the study constructs into relatively high to low by their corresponding importance and performance of the endogenous construct [23]. IMPA distinguishes the possible area of improvement from the managerial and literature perspective. IPMA analysis transforms the total effect of the rescaled variables totals in the un-standardised technique [25]. Rescaling is recognised for every latent constructs’ score between 0 and 100. The mean value of the latent variable score represents the performance of the latent variable, where 0 indicates the least and 100 indicates the maximum importance in the performance of the endogenous construct [20].

## 4. Data Analysis

### 4.1. Demographic Characteristics

As Presented in Table 1, the data were collected from mostly females (57.4%). The following are the percentage for age: below 21 years old (28.4%), between 21–25 years old (57.5%), between 26–30 years old (7.7%), of between 31–35 years old (2.4%), and the remaining respondents are 36–40 years old. The majority of the respondents are single (93.4%), and the remaining respondents are married or divorced. The majority of the respondents completed their bachelor’s degree or equivalent (60.6%). The following are the percentage for education level: secondary school level (17.6%), diploma or technical school level (19.7%), master’s level (1.8%), and the remaining respondents completed their doctoral-level education. The following are the percentage for monthly income: less than RM2500 (75.3%), between RM2501–RM5000 (17%), between RM5001–RM7500 (4.5%), between RM7501–RM10,000 (1.5%), and the remaining respondents have an income of more than RM10,000. The majority of the study respondents live in urban areas (89.2%). The most significant segment of the respondents are of Chinese origin (88.9%), followed by other origins (6.1%), Malaysian (2.8%), and Indian origin (2.2%).

### 4.2. Reliabilities and Validities

Following the approval of Hair et al. [20], the reliabilities for study’s latent constructs can be achieved and assessed by Cronbach’s alpha (CA), DG rho, and composite reliability (CR). Cronbach’s alpha values for each construct are above the threshold of 0.70, and the minimum value of Cronbach’s alpha value achieves 0.781 [23]. The results are reported in Table 2. Furthermore, all DG rho values are above the threshold of 0.70, where the minimum value of DG rho is 0.783 [20]. Moreover, CR values are well beyond the threshold of 0.70, where the lowest value of CR value is 0.850 [23]. These outcomes indicate that the latent constructs realised the suitable reliabilities, and they performed well for the later stage of analysis. AVE for all items for each construct must be above 0.50 score to the extent the adequate convergent validity to support the uni-dimensionality concept for each construct [20]. Items display that the constructs have acceptable convergent validity (see Table 2.). All the VIF values for each construct are below the threshold of 3.3 that reveals no concern of multicollinearity [23]. The item loading and cross-loading for the confirmation of construct discriminant validity are described in Table 3 and Table 4, respectively.

All the study constructs have appropriate discriminant validities (see Table 3). Additionally, the Fornell–Larcker criterion (1981) and HTMT ratio had achieved the discriminant validity of each study construct. The Fornell–Larcker criterion was assessed with the square root of the respective construct’s AVE, and the square root of AVE for the construct must be higher than the correlation among other constructs [20]. HTMT ratio needs to be less than 0.85 to establish discriminant validity for each study construct [26]. Table 3 and Table 4 show that the study has adequate discriminant validity for each construct.

### 4.3. Path Analysis

The reliabilities and validities from the structural assessment of the study model are satisfactory. The next measurement assessment examined the study hypothesis. The adjusted *r^2^* value for the five exogenous constructs (i.e., HTC, KHF, ATF, SUN, and PBC)) on IHF explains the 50.3% change in the intention to consume healthy food. The predictive relevance (Q^2^) value for the part of the model is 0.343, indicating a large predictive relevance [23]. The adjusted *r^2^* value for the exogenous construct (i.e., intention to consume healthy food) on the CHF elucidates 8.2% change in the CHF. The predictive relevance (Q^2^) value for the part of the model is 0.078, indicating small predictive relevance [23].

Model standardised path values, t-values, and significance level are illustrated in Table 5. The path coefficient between HTC and IHF (β = 0.344, *t* = 10.825, *p* = 0.000) indicates a significant and positive effect of HTC on the intention to consume healthy food. The result forms significant statistical support for H1. The path value for KHF and IHF (β = 0.203, *t* = 6.556, *p* = 0.000) shows the impact of KHF for the intention to consume healthy food, which is positive and significant; hence, it offers significant statistical support for H2. The path between AHF and IHF (β = 0.109, *t* = 4.289, *p* = 0.000) shows the influence of AHF in influencing the intention to consume healthy food, which is positive and significant; it supports H3. The path coefficient for SBN and IHF (β = 0.076, *t* = 2.815, *p* = 0.003) shows a positive and significant effect; it supports H4. The path between PBC and IHF (β = 0.102, *t* = 3.381, *p* = 0.000) shows the influence of PBC in influencing the intention to consume healthy food, which is positive and significant; it supports H5. The path coefficient for IHF and CHF (β = 0.267, *t* = 11.570, *p* = 0.000) shows a positive and significant effect; it supports H6. Table 5 shows the path coefficients.

### 4.4. Mediation Analysis

The mediation effect of IHF was tested with H_7A_ for the relationship between HTC and CHF. The result reveals that IHF mediates the relationship between HTC and CHF (β = 0.092, CI min = 0.072, CI max = 0.112, *p* = 0.000) and supports H_7A_. For H_7B_, the relationship between KHF and CHF is mediated by IHF. The result shows that IHF mediates the relationship between KHF and CHF (β = 0.054, CI min = 0.039, CI max = 0.071, *p* = 0.000); it supports H_7B_. For H_7C,_ the relationship between AHF and CHF is mediated by IHF. The result shows that IHF mediates the relationship between AHF and CHF (β = 0.029, CI min = 0.017, CI max = 0.041, *p* = 0.000); it supports H_7C_. For H_7D,_ the relationship between SBN and CHF is mediated by IHF. The result reveals that IHF mediates the relationship between SBN and CHF (β = 0.020, CI min = 0.008, CI max = 0.033, *p* = 0.004); it supports H_7D_. For H_7E_, the relationship between PBC and CHF is mediated by IHF. The result reveals that IHF mediates the relationship between PBC and CHF (β = 0.027, CI min = 0.014, CI max = 0.042, *p* = 0.001); it supports H_7E_. The mediation results are presented in Table 6.

### 4.5. Multi-Group Analysis

Multi-group analyses were executed to match the results for different groups based on gender, living area, and education. One non-parametric test was employed to evaluate the differences in the vital association between the model based on gender, areas of living, and education of the sample. Table 7 shows the path values for two groups with the differences within the groups with the *p*-values as recommended by Henseler et al. [24]. P_MGA_ represents the p-values using the multi-group analysis of PLS-SEM as the measure for the significance of the difference between groups [24].

#### 4.5.1. Effects of Gender

The results of the groups are based on gender in the sample. Gender shows no significant difference in the relationships of the model. The variance of gender does not influence the relationship between study models.

#### 4.5.2. Effects of Living Area

The results of the two groups are based on the living area—namely, urban and rural. Living area shows a significant difference in the relationship between PBS and CHF, IHF, and CHF for CHF. Living area does not influence the variance between the model’s other paths.

#### 4.5.3. Effects of Education

The results of the two groups are based on the education of the sample. The variance of education does not influence the variance between the study’s paths.

#### 4.5.4. Effects of Household Income

The results of the three groups (below RM2500, between RM2501–RM5000; below RM2500 and between RM5001–RM7500; RM2501–RM5000 and RM5001–RM7500) presented in Table 7 are based on the respondents’ household income. The findings revealed a significant difference in the relationship between the effect of AHF on IHF, and PBC on IHF among the respondents with a household income below RM2500 and between RM2501 and RM5000. The findings also showed a significant difference in the relationship between the effect of PBC on IHF among respondents with a household income between RM2501–RM5000 and RM5001–RM7500.

### 4.6. Importance Performance Matrix

**Figure 2:**
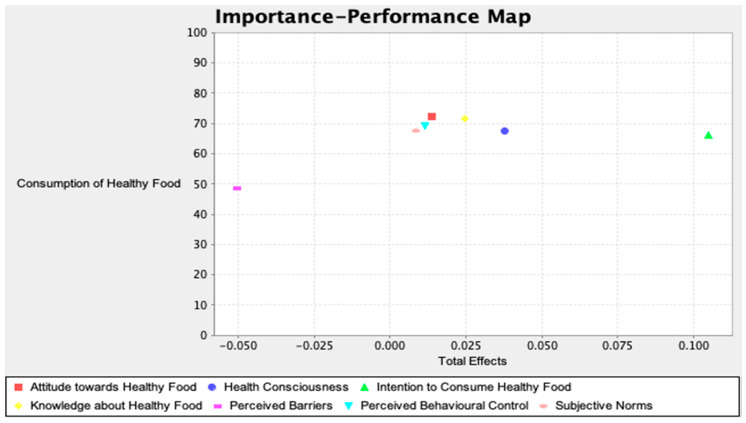
Importance performance map.

Figure 2 and Table 8 shows the outcomes of the IPMA, and it displays that ATF is the most vital cause in the performance of CHF (0.109; 72.177), followed by KHF (0.203; 71.551), PBC (0.102, 69.109), and SBN (0.076; 67.507).

## 5. Discussion

The first five hypotheses evaluated the effects of HTC, KHF, AHF, SBN, and PBC on IHF. The study findings support the argument that HTC (*f*^2^ =0.110) has a medium effect on IHF, and KHF (*f*^2^ =0.032) has a small effect on IHF. However, the effects of AHF (*f*^2^ =0.012), SBN (*f*^2^ =0.006), and PBC (*f*^2^ = 0.010) have a significant but small effect on IHF among Malaysian young adults [23]. Study findings are parallel to the findings by Hoque et al. [4] that HTC and knowledge influence the intention to consume healthy food. HTC and food knowledge were also found to significantly influence intention in developing countries as well [8]. Furthermore, the findings from the study revealed that AHF, SBN, and PBC affected IHF, which correspond with the results in a study by Menozzi et al. [5]. However, the effect sizes of the AHF, SBN, and PBC on IHF were significant but below the small effect threshold compared to the results of Menozzi et al. [5]. Accordingly, this indicates the low level of AHF, SBN and PBC among the Malaysian respondents in having the intention to consume healthy food.

The next hypotheses proposed the effects of PBS and IHF on CHF. The study findings support the argument that PBS (*f*^2^ =0.019) has a small effect on CHF, match with the results reported by Nguyen et al. [13] in which the influences of PBS are both significant and negative regarding the use of green products. The results of our study also suggest a similar pattern in that PBS negatively influences the CHF and reduces the CHF among the study sample. However, the effect of IHF (*f*^2^ =0.077) has a small, positive, and significant effect on CHF [23]. Although the findings from our study are comparable to those claimed by Menozzi et al. [5] and Maichum et al. [27] in which intention significantly and positively affects consumption behaviour.

The next mediating effect of IHF was assessed with five mediation hypotheses. H_7A_ investigated the mediating effect of IHF between HTC and CHF. The finding approves the meditating effect of IHF (β = 0.092, *p* = 0.000) for the relationship between HTC and CHF among Malaysian young adults for the CHF. The findings of this study support several studies [15,27]. H_7B_ hypothesised about the meditating effect of IHF between KHF and CHF. The finding confirms the meditating effect of IHF (β = 0.054, *p* = 0.000) for the relationship between KHF and CHF for the healthy food consumption among Malaysian young adults. The finding of this study is supported by Maichum et al. [27].

The next hypothesis, H_7C_, evaluated the meditating effect of IHF between AHF and CHF. The finding confirms that the significant mediating effect of IHF (β = 0.029, *p* = 0.000) for the relationship between AHF and CHF. The study results are supported by Yadav and Pathak [15]. Furthermore, H_7D_ estimated the meditating effect of IHF between the relationship of SBN and CHF. The finding confirms the mediating effect of IHF (β = 0.020, *p* = 0.004) for the relationship between SBN and CHF. The study results are supported by Yadav and Pathak [15]. H_7E_ assessed the mediating effect of IHF between PBC and CHF. The finding confirms the meditating effect of IHF (β = 0.027, *p* = 0.001) for the relationship between PBC and CHF. Further, IHF significantly mediates between all the factors (i.e., HTC, KHF, AHF, SBN, and PBC) and relationships with the CHF, whereby intention significantly enhances the relationship for the subject factors on the CHF.

The moderating effect of PBS was evaluated for the relationship between IHF and CHF. Study findings suggest that PBS significantly moderates the relationship between IHF and CHF. The perception of barriers reduces CHF. However, the moderating effect of PBS had a reduced effect on the relationship between the IHF and CHF. Moreover, high intention reduced the effect of PBS for CHF. However, PBS needs to be contained so as to increase the consumption behaviour for healthy foods [13]. Our study is pioneering in testing the moderating effect of PBS for the relationship between IHF and CHF and is therefore important to understand that consumers having high intention felt less about PBS than CHF and vice versa.

The multiple-group analysis estimated the effect of respondents’ personal features of gender, residence area, and education. The PLS multi-group analysis technique investigated the effects of respondents’ characteristics. Study results reveal no significant variance for respondents’ gender on the study paths, and there is no significant difference between study paths based on gender. There is a significant difference between PBS and CHF for the respondents’ living area—namely, urban and rural areas. There is a significant difference in the path between IHF and CHF. However, there is no significant difference for other paths and no significant difference based on respondents’ area of living. Moreover, there is no significant difference for other paths of the study model based on the respondents’ education. Multigroup analysis also revealed that the effect of AHF on IHD was significantly higher among the lower-income group compared to the higher income group. Moreover, the effect of PBC on IHF was much lower among the middle-income group than that of the other two groups.

Subsequently, this study estimated the performance of CHF with the factors of HTC, KHF, AHF, SBN, PBC, PBS, and IHF. The most critical three factors for the performance for CHF are AHF, KHF, and PBC. Besides, the fourth and fifth most important factors for the performance of CHF are HTC and SBN for the CHF.

## 6. Conclusions

It is important to have healthy nations, and the health of a nation depends on healthy food consumption by the youth of that nation [3]. The current study explored the effect of HTC and KHF that impact the IHF by factors of attitude, SUN, and PBC. The study also included the factor of PBS for healthy food in influencing CHF among Malaysian young adults.

Young people around the world have significant consumers at a global level [28]. The young Malaysian population is increasingly interested in having a healthy lifestyle and getting involved in healthy food consumption [3]. Healthy eating is increased with the personal pro-health behaviours, and it is affected by the PBS for healthy food products [4]. Global youth is encouraged to get engaged in pro-social and personal health-related consumption [29].

Study findings have several implications in developing effective strategies for healthy food consumption. The effects of HTC, KHF, AHF, SUN, and PBC positively influence IHF among Malaysian young adults. Attitude is the most significant contributor to the intention to consume healthy food. Marketers and government agencies must increase the information and promotion of healthy food [3]. It helps to enhance the level of information and knowledge of general consumers and also helps to promote healthy eating habits [12], as government intervention can ensure the reduced prices for healthy food. KHF is important for the intention to consume healthy food. CHF is significantly reduced by PBS. PBS needs to be controlled by the provision of healthy foods at superstores. Reduced prices, availability, and general consumer attitude toward healthy food can also aid in addressing the issue of obesity and empower the public to lead a healthy lifestyle [10]. The information and promotional activities need to be activated to enhance awareness and influence knowledge and consciousness of healthy food.

The study has the following three limitations. The study analysis was performed on the cross-sectional data that have limited generalisability. Future research should consider the longitudinal data to understand the time lag between IHF and CHF. However, the study model can be utilised to explore the consumption of organic food. PBS can be utilised to understand the restricting factors for CHF among study samples. PBS is higher among urban respondents than rural samples. Future studies can explore the factors to tackle the PBS in improving the CHF. This study contributes to the healthy food adoption model by adding the factor of PBS. Future research can evaluate the role of different barriers for IHF. The current study estimated that the general perception of healthy food consumption and knowledge of healthy food is inconsistent and requires further investigation [6]. This may be seen as a further limitation in generalising the findings of this study to a wider population. However, general knowledge of consumers regarding the influence of healthy food is a social and environmental concern [3]. In this regard, future studies could use specific knowledge of healthy food in establishing the intention and behaviour of consumers toward a vast range of healthy food products.

## Figures and Tables

**Table 1:** Demographic characteristics.

	*N*	%		*N*	%
*Gender*			*Marital Status*		
Male	704	42.6	Single	1542	93.4
Female	947	57.4	Married	100	6.1
Total	1651	100.0	Divorced	6	0.4
			Total	1651	100
*Age Group*					
Below 21 years	469	28.4	*Education*		
21–25 years	950	57.5	Secondary school certificate	291	17.6
26–30 years	127	7.7	Diploma/technical school certificate	325	19.7
31–35 years	40	2.4	Bachelor’s degree or equivalent	1001	60.6
36–40 years	65	3.9	Master’s degree	30	1.8
Total	1651	100.0	Doctoral degree	4	0.2
			Total	1651	100.0
*Ethnicity*					
Malay	47	2.8	*Household Income*		
Chinese	1467	88.9	Below RM2500	1244	75.3
Indian	36	2.2	RM2501–RM5000	281	17.0
Others	101	6.1	RM5001–RM7500	74	4.5
Total	1651	100.0	RM7501–RM10,000	25	1.5
			RM10,001–RM12,500	15	0.9
*Living Areas*			More than RM12,500	12	0.7
Rural	178	10.8	Total	1651	100.0
Urban	1473	89.2			
Total	1651	100.0			

**Table 2:** Reliability and validity.

Variables	No. Items	Mean	SD	CA	DG *rho*	CR	AVE	VIF
HTC	6	5.421	0.839	0.890	0.891	0.916	0.646	2.162
KHF	6	5.063	0.960	0.806	0.818	0.860	0.507	2.613
AHF	5	5.135	0.954	0.781	0.783	0.850	0.531	2.005
SBN	6	5.305	0.882	0.823	0.831	0.871	0.531	1.841
PBC	6	5.046	0.984	0.827	0.835	0.873	0.535	2.199
IHF	6	3.909	1.232	0.909	0.910	0.929	0.687	1.013
PBS	6	4.970	1.0278	0.862	0.896	0.896	0.590	1.346
CHF	1	0.800	0.404	1.000	1.000	1.000	1.000	-

Note: HTC: health consciousness; KHF: knowledge about healthy food; AHF: attitude toward healthy food; SBN: subjective norms; PBC: perceived behavioural control; IHF: intention to consume healthy food; PBS: perceived barriers; CHF: consumption of healthy food; SD: standard deviation; CA: Cronbach’s alpha; D.G. *rho*: Dillo–Goldstein’s *rho*; CR: composite reliability; AVE: average variance extracted; VIF—variance inflation factors. Source: authors’ data analysis.

**Table 3:** Discriminant validity.

	HTC	KHF	AHF	SBN	PBC	IHF	PBS	CHF
*Fornell–Larcker Criterion*								
HTC	0.804							
KHF	0.660	0.712						
AHF	0.608	0.623	0.729					
SBN	0.580	0.589	0.574	0.728				
PBC	0.599	0.700	0.576	0.545	0.732			
IHF	0.650	0.614	0.547	0.513	0.554	0.829		
PBS	0.035	−0.089	−0.084	0.080	−0.052	0.065	0.768	
CHF	0.243	0.221	0.226	0.195	0.207	0.254	−0.105	1.000
*Heterotrait–Monotrait Ratio (HTMT)*								
HTC	-							
KHF	0.764	-						
AHF	0.709	0.776	-					
SBN	0.670	0.713	0.705	-				
PBC	0.684	0.856	0.694	0.647	-			
IHF	0.722	0.703	0.637	0.585	0.625	-		
PBS	0.095	0.099	0.114	0.120	0.140	0.111	-	
CHF	0.258	0.237	0.247	0.211	0.221	0.266	0.107	-

Note: HTC: health consciousness; KHF: knowledge about healthy food; AHF: attitude toward healthy food; SBN: subjective norms; PBC: perceived behavioural control; IHF: intention to consume healthy food; PBS: perceived barriers; CHF: consumption of healthy food. Source: authors’ data analysis.

**Table 4:** Loadings and cross-loading.

Code	HTC	KHF	AHF	SBN	PBC	IHF	PBS	CHF
HTC-Item 1	*0.780*	0.556	0.495	0.470	0.474	0.525	−0.026	0.221
HTC-Item 2	*0.822*	0.524	0.507	0.454	0.480	0.521	0.032	0.234
HTC-Item 3	*0.808*	0.526	0.477	0.449	0.459	0.514	0.048	0.161
HTC-Item 4	*0.826*	0.545	0.517	0.473	0.507	0.549	0.013	0.178
HTC-Item 5	*0.782*	0.509	0.454	0.476	0.474	0.490	0.089	0.190
HTC-Item 6	*0.805*	0.524	0.478	0.479	0.492	0.531	0.018	0.189
KHF-Item 1	0.400	*0.634*	0.434	0.365	0.654	0.370	−0.091	0.083
KHF-Item 2	0.557	*0.734*	0.475	0.477	0.547	0.503	−0.073	0.237
KHF-Item 3	0.455	*0.749*	0.457	0.388	0.458	0.413	−0.074	0.121
KHF-Item 4	0.562	*0.760*	0.502	0.480	0.497	0.540	−0.060	0.196
KHF-Item 5	0.388	*0.666*	0.355	0.383	0.382	0.348	−0.013	0.139
KHF-Item 6	0.407	*0.721*	0.414	0.393	0.455	0.398	−0.065	0.133
AHF-Item 1	0.346	0.447	*0.729*	0.372	0.352	0.356	−0.110	0.141
AHF-Item 2	0.370	0.462	*0.766*	0.406	0.359	0.354	−0.096	0.125
AHF-Item 3	0.406	0.485	*0.758*	0.426	0.382	0.374	−0.042	0.122
AHF-Item 4	0.458	0.421	*0.668*	0.418	0.486	0.391	−0.041	0.169
AHF-Item 5	0.579	0.451	*0.720*	0.452	0.484	0.483	−0.030	0.238
SBN-Item 1	0.376	0.398	0.378	*0.731*	0.354	0.331	0.099	0.085
SBN-Item 2	0.335	0.429	0.402	*0.668*	0.342	0.300	−0.030	0.135
SBN-Item 3	0.460	0.383	0.395	*0.688*	0.415	0.366	0.084	0.155
SBN-Item 4	0.474	0.452	0.461	*0.792*	0.422	0.444	0.094	0.164
SBN-Item 5	0.383	0.426	0.398	*0.747*	0.364	0.354	0.023	0.134
SBN-Item 6	0.478	0.480	0.465	*0.738*	0.466	0.418	0.058	0.166
PBC-Item 1	0.491	0.543	0.491	0.440	*0.727*	0.510	−0.071	0.183
PBC-Item 2	0.486	0.481	0.425	0.408	*0.767*	0.413	−0.037	0.208
PBC-Item 3	0.299	0.491	0.367	0.334	*0.690*	0.318	−0.081	0.084
PBC-Item 4	0.433	0.489	0.399	0.389	*0.775*	0.376	−0.032	0.179
PBC-Item 5	0.477	0.474	0.398	0.432	*0.718*	0.403	0.074	0.139
PBC-Item 6	0.395	0.591	0.417	0.362	*0.707*	0.362	−0.087	0.089
IHF-Item 1	0.501	0.491	0.412	0.389	0.433	*0.778*	0.056	0.181
IHF-Item 2	0.529	0.542	0.465	0.416	0.470	*0.808*	0.029	0.172
IHF-Item 3	0.569	0.491	0.467	0.428	0.455	*0.840*	0.070	0.240
IHF-Item 4	0.538	0.498	0.444	0.430	0.471	*0.857*	0.059	0.235
IHF-Item 5	0.537	0.518	0.455	0.446	0.458	*0.844*	0.073	0.216
IHF-Item 6	0.554	0.516	0.473	0.442	0.469	*0.843*	0.034	0.216
PBS-Item 1	0.070	−0.040	−0.036	0.116	0.039	0.048	*0.725*	−0.048
PBS-Item 2	0.063	−0.037	−0.049	0.063	−0.107	0.049	*0.756*	−0.068
PBS-Item 3	−0.061	−0.131	−0.142	0.033	−0.061	−0.051	*0.840*	−0.113
PBS-Item 4	0.008	−0.058	−0.048	0.004	−0.127	0.059	*0.757*	−0.078
PBS-Item 5	0.084	−0.056	−0.018	0.116	0.036	0.127	*0.738*	−0.068
PBS-Item 6	0.065	−0.047	−0.044	0.079	0.014	0.119	*0.790*	−0.083
CHF-Item 1	0.243	0.221	0.226	0.195	0.207	0.254	−0.105	*1.000*

Note: HTC: health consciousness; KHF: knowledge about healthy food; AHF: attitude toward healthy food; SBN: subjective norms; PBC: perceived behavioural control; IHF: intention to consume healthy food; PBS: perceived barriers; CHF: consumption of healthy food; (2) Values in italics in the matrix above are the item loadings and others are cross-loadings. Source: authors’ data analysis.

**Table 5:** Path coefficients.

Hypo		Beta	CI-Min	CI-Max	*t*	*p*	*r* ^2^	*f^2^*	Q^2^	Decision
*Factors affecting the Intention to Consume Healthy Food*										
H_1_	HTC ➔ IHF	0.344	0.288	0.395	10.825	0.000		0.110		Accept
H_2_	KHF ➔ IHF	0.203	0.153	0.256	6.556	0.000		0.032		Accept
H_3_	AHF ➔ IHF	0.109	0.066	0.150	4.289	0.000	0.503	0.012	0.343	Accept
H_4_	SBN ➔ IHF	0.076	0.031	0.124	2.815	0.003		0.006		Accept
H_5_	PBC ➔ IHF	0.102	0.052	0.153	3.381	0.000		0.010		Accept
*Factor affecting the Consumption of Healthy Food*										
H_6_	IHF ➔ CHF	0.267	0.225	0.304	11.570	0.000	0.082	0.077	0.078	Accept
*Moderating Effect of Perceived Barriers*										
	PBS ➔ CHF	−0.155	−0.205	−0.112	5.414	0.000		0.019		
H_8_	IHF ➔ CHF	0.055	0.014	0.099	2.206	0.014			Moderation	

Note: HTC: health consciousness; KHF: knowledge about healthy food; AHF: attitude toward healthy food; SBN: subjective norms; PBC: perceived behavioural control; IHF: intention to consume healthy food; PBS: perceived barriers; CHF: consumption of healthy food. Source: authors’ data analysis.

**Table 6:** Mediating effects.

Hypo	Associations	Beta	CI-Min	CI-Max	*t*	*p*	Decision
H_7A_	HTC ➔ IHF ➔ CHF	0.092	0.072	0.112	7.767	0.000	Accept
H_7B_	KHF ➔ IHF ➔ CHF	0.054	0.039	0.071	5.640	0.000	Accept
H_7C_	AHF ➔ IHF ➔ CHF	0.029	0.017	0.041	4.027	0.000	Accept
H_7D_	SBN ➔ IHF ➔ CHF	0.020	0.008	0.033	2.699	0.004	Accept
H_7E_	PBC ➔ IHF ➔ CHF	0.027	0.014	0.042	3.289	0.001	Accept

Note: HTC: health consciousness; KHF: knowledge about healthy food; AHF: attitude toward healthy food; SBN: subjective norms; PBC: perceived behavioural control; IHF: intention to consume healthy food; PBS: perceived barriers; CHF: consumption of healthy food. Source: authors’ data analysis.

**Table 7:** Multi-group analysis.

	Male		Female		Difference		
	Beta	*p*-Value	Beta	*p*-Value	Beta	*p*-Value	Decision
HTC ➔ IHF	0.392	0.000	0.304	0.000	0.088	0.080	No Difference
KHF ➔ IHF	0.170	0.000	0.229	0.000	−0.059	0.174	No Difference
AHF ➔ IHF	0.106	0.003	0.103	0.001	0.003	0.475	No Difference
SBN ➔ IHF	0.118	0.001	0.050	0.075	0.068	0.102	No Difference
PBC ➔ IHF	0.062	0.076	0.139	0.000	−0.076	0.086	No Difference
IHF ➔ CHF	0.285	0.000	0.257	0.000	0.028	0.267	No Difference
PBS ➔ CHF	−0.158	0.001	−0.159	0.000	0.001	0.481	No Difference
IHF ➔ CHF (Moderating)	0.039	0.161	0.075	0.006	−0.036	0.231	No Difference
	**Urban**		**Rural**		**Difference**		
	**Beta**	***p*-Value**	**Beta**	***p*-Value**	**Beta**	***p*-Value**	**Decision**
HTC ➔ IHF	0.257	0.002	0.350	0.000	−0.093	0.161	No Difference
KHF ➔ IHF	0.126	0.054	0.216	0.000	−0.090	0.139	No Difference
AHF ➔ IHF	0.207	0.003	0.096	0.000	0.111	0.075	No Difference
SBN ➔ IHF	0.074	0.148	0.078	0.002	−0.004	0.479	No Difference
PBC ➔ IHF	0.208	0.004	0.090	0.001	0.118	0.073	No Difference
IHF ➔ CHF	0.264	0.000	0.263	0.000	0.001	0.482	No Difference
PBS ➔ CHF	−0.295	0.000	−0.150	0.000	−0.145	0.032	Sig. Difference
IHF ➔ CHF (Moderating)	0.167	0.006	0.047	0.030	0.119	0.047	Sig. Difference
	**Secondary School Certificate**		**Bachelor’s Degree or Equivalent**		**Difference**		
	**Beta**	***p*-Value**	**Beta**	***p*-Value**	**Beta**	***p*-Value**	**Decision**
HTC ➔ IHF	0.243	0.003	0.378	0.000	−0.135	0.065	No Difference
KHF ➔ IHF	0.304	0.001	0.202	0.000	0.101	0.152	No Difference
AHF ➔ IHF	0.049	0.213	0.119	0.000	−0.069	0.158	No Difference
SBN ➔ IHF	0.046	0.246	0.070	0.017	−0.025	0.367	No Difference
PBC ➔ IHF	0.157	0.022	0.066	0.024	0.091	0.144	No Difference
IHF ➔ CHF	0.183	0.001	0.268	0.000	−0.085	0.093	No Difference
PBS ➔ CHF	−0.177	0.038	−0.161	0.000	−0.015	0.318	No Difference
IHF ➔ CHF (Moderating)	0.091	0.055	0.075	0.007	0.016	0.385	No Difference
	**Income** **(Below RM2500)**		**Income** **(RM2501–RM5000)**		**Income** **(RM5001–RM7500)**		
	**Beta**	***p*-Value**	**Beta**	***p*-Value**	**Beta**	***p*-Value**	
HTC ➔ IHF	0.336	0.000	0.386	0.000	0.387	0.004	
KHF ➔ IHF	0.201	0.000	0.268	0.000	0.112	0.254	
AHF ➔ IHF	0.139	0.000	0.009	0.438	−0.034	0.404	
SBN ➔ IHF	0.067	0.007	0.094	0.134	0.094	0.254	
PBC ➔ IHF	0.113	0.000	−0.033	0.341	0.319	0.020	
IHF ➔ CHF	0.267	0.000	0.288	0.000	0.227	0.009	
PBS ➔ CHF	−0.170	0.000	−0.148	0.052	−0.167	0.265	
IHF ➔ CHF (Moderating)	0.068	0.011	0.034	0.283	0.108	0.276	
	**Difference (Below RM2500 VS RM2501–RM5000)**		**Difference (Below RM2500 VS RM5001–RM7500)**		**Difference (RM2501–RM5000 VS RM5001–RM7500)**		
	**Beta**	***p*-Value**	**Beta**	***p*-Value**	**Beta**	***p*-Value**	**Decision**
HTC ➔ IHF	−0.049	0.286	−0.051	0.355	−0.001	0.489	No Difference
KHF ➔ IHF	−0.067	0.216	0.089	0.303	0.156	0.208	No Difference
AHF ➔ IHF	0.130	0.026	0.173	0.120	0.043	0.369	Sig. Difference
SBN ➔ IHF	−0.027	0.385	−0.027	0.432	0.000	0.493	No Difference
PBC ➔ IHF	0.146	0.047	−0.207	0.088	−0.352	0.020	Sig. Difference
IHF ➔ CHF	−0.020	0.373	0.041	0.342	0.061	0.292	No Difference
PBS ➔ CHF	−0.022	0.444	−0.003	0.342	0.019	0.341	No Difference
IHF ➔ CHF (Moderating)	0.034	0.309	−0.040	0.365	−0.074	0.332	No Difference

Note: HTC: health consciousness; KHF: knowledge about healthy food; AHF: attitude toward healthy food; SBN: subjective norms; PBC: perceived behavioural control; IHF: intention to consume healthy food; PBS: perceived barriers; CHF: consumption of healthy food. Source: authors’ data analysis.

**Table 8:** Performance and total effects.

Target Construct	Consumption of Healthy Food	
Variables	Total Effect	Performance
Health Consciousness	0.344	67.481
Knowledge about Healthy Food	0.203	71.551
Attitude towards Healthy Food	0.109	72.177
Subjective Norms	0.076	67.507
Perceived Behavioural Control	0.102	69.109
Intention to Consume Healthy Food	0.267	66.175
Perceived Barriers	−0.155	48.440
Consumption of Healthy Food	-	79.528

Source: authors’ data analysis.

## Appendix A. Survey Instrument

**Code**	**Items**
HTC-Item 1	I choose food carefully to ensure good health
HTC-Item 2	I consider myself as a health-conscious consumer
HTC-Item 3	I often think about health-related issues
HTC-Item 4	I am prepared to do anything that is good to health
HTC-Item 5	I often dwell on my health
HTC-Item 6	I think that I take health into account a lot in my life
KHF-Item 1	I am familiar with healthy foods
KHF-Item 2	I am knowledgeable about the impact of unhealthy foods
KHF-Item 3	I am interested in finding out more about healthy foods
KHF-Item 4	I am informed that the healthy foods contain fewer harmful chemicals than unhealthy foods
KHF-Item 5	I am informed that the consumption of unhealthy food is harmful for health
KHF-Item 6	Reading of production and expiration date on food package is important
AHF-Item 1	Consuming healthy foods will improve my overall health
AHF-Item 2	Consuming healthy foods can prevent and reduce the risk of specific health conditions
AHF-Item 3	Consuming healthy foods is a preventive measure for certain illness
AHF-Item 4	Consuming healthy foods per day is not difficult
AHF-Item 5	Consuming healthy foods is in line with my food style
SBN-Item 1	My friends or colleagues think I should consume healthy foods
SBN-Item 2	My family expects me to consume healthy foods
SBN-Item 3	Most people I value would buy healthy foods
SBN-Item 4	Most friends whose opinions regarding diet are important to me think that I should buy healthy foods
SBN-Item 5	My doctor thinks I should consume healthy foods
SBN-Item 6	The media encouragements make me think the best way one could become healthy is to consume healthy foods
PBC-Item 1	If I wanted to, I could buy healthy foods instead of non- healthy foods.
PBC-Item 2	I think it’s easy for me to buy healthy foods
PBC-Item 3	It’s mostly up to me whether or not to buy healthy foods
PBC-Item 4	I have resources, time and opportunities to buy healthy foods
PBC-Item 5	I am confident that if I want, I can buy healthy foods at place of conventional unhealthy foods
PBC-Item 6	Whether I consume healthy foods is a decision that depends entirely on me
IHF-Item 1	I want to purchase healthy foods if they are available for purchase.
IHF-Item 2	I want to consume healthy foods if they available for purchase.
IHF-Item 3	I intend to consume at least two servings of healthy foods per day
IHF-Item 4	I intend to consume at least two servings healthy foods to have a balanced diet
IHF-Item 5	I intend to consume at least two servings healthy foods to protects me from being diagnosed with any medical condition
IHF-Item 6	I intend to consume at least two servings healthy foods to protects me from harming my health
PBS-Item 1	I do not like the smell of natural healthy foods
PBS-Item 2	It is not convenient for me to purchase healthy foods
PBS-Item 3	I do not like the taste of healthy foods
PBS-Item 4	I cannot afford to pay more to healthy foods
PBS-Item 5	While shopping, I can’t easily distinguish between healthy foods and unhealthy foods
PBS-Item 6	I am not confident about the credibility of healthy foods
CHF-Item 1	I have been eaten at least two servings healthy foods last week
